# Off-Clamp Versus On-Clamp Partial Nephrectomy: An Updated Systematic Review, Meta-Analysis and Meta-Regression

**DOI:** 10.3390/jcm15072792

**Published:** 2026-04-07

**Authors:** Paweł Dębiński, Jakub Karwacki, Łukasz Nowak, Zuzanna Szczepaniak, Maria Jędryka, Karol Zagórski, Bartosz Małkiewicz, Tomasz Szydełko

**Affiliations:** 1 Department of Urology, University Centre of Excellence in Urology, Wroclaw Medical University, ul. Borowska 213, 50-556 Wroclaw, Poland; 2 Department of Minimally Invasive and Robotic Urology, University Centre of Excellence in Urology, Wroclaw Medical University, ul. Borowska 213, 50-556 Wroclaw, Poland

**Keywords:** ischemia, kidney cancer, partial nephrectomy, renal function

## Abstract

**Objectives:** The impact of renal ischemia during partial nephrectomy (PN) on postoperative renal function remains controversial. On-clamp PN provides improved surgical exposure and haemostasis but induces warm ischemia, which may impair renal function. Off-clamp PN avoids ischemia-related injury and may better preserve renal function, although concerns persist regarding blood loss and oncological safety. We systematically compared perioperative and functional outcomes, as well as surgical margin status between on-clamp and off-clamp PN. **Methods:** We performed a systematic search of PubMed, Embase, Cochrane, Web of Science, and Scopus to identify randomized controlled trials (RCTs) and observational studies comparing on-clamp versus off-clamp PN with no publication time limitations. Outcomes included estimated glomerular filtration rate (eGFR), percentage eGFR change, estimated blood loss (EBL), transfusion rates, positive surgical margins (PSMs), operative time, and complications. **Results:** Thirty-nine studies (four RCTs) including 10,154 patients were analysed. Off-clamp PN was associated with a smaller decline in eGFR (mean difference [MD] −4 mL/min/1.73 m^2^, 95% CI −5.7 to −2.8) and lower percentage eGFR loss (MD −1.7%, 95% CI −2.8 to −0.7). On-clamp PN was associated with lower EBL (MD −48 mL, 95% CI −72 to −25). Transfusion rates favored on-clamp PN but were not statistically significant (OR 0.7, 95% CI 0.5–1.0). On-clamp PN was associated with a higher risk of PSM (OR 1.3, 95% CI 1.0–1.7) and postoperative complications (OR 1.3, 95% CI 1.1–1.6). Between-study heterogeneity and predominance of observational data were key limitations. **Conclusions:** Off-clamp PN provides superior renal functional preservation and lower risks of PSMs and complications, at the cost of increased blood loss. These findings support individualized surgical decision-making based on patient and tumor characteristics. What does the study add?: This study provides an extensive and detailed comparison of off-clamp versus on-clamp partial nephrectomy, encompassing more than 10,000 patients from 39 studies. By integrating the available evidence up to late 2024, it delivers comprehensive estimates of the renal functional benefits associated with ischemia-free surgery. Our findings delineate the trade-offs between renal preservation, blood loss, and surgical margin status, thereby informing individualised decision-making in nephron-sparing surgery and refining current understanding of when ischemia avoidance is most clinically advantageous. Patient summary: Our study suggests that performing partial nephrectomy without temporarily clamping the kidney blood vessels may better preserve kidney function and reduce cancer-related surgical risks, but can lead to increased blood loss during surgery. These findings indicate that the choice of surgical technique should be individualised, taking into account tumour features and patient-specific factors.

## 1. Introduction

Partial nephrectomy (PN) is the gold standard for localized renal tumours, aiming to preserve renal function while achieving oncological control [1]. Traditionally, hilar clamping has been used to minimize intraoperative bleeding, but at the cost of warm ischemia and potential renal parenchymal injury [2]. Off-clamp PN avoids ischemia but may increase intraoperative blood loss and potentially compromise margin status [3].

Previous randomized controlled trials (RCTs) and systematic reviews have shown conflicting results regarding the superiority of one technique over the other [4,5]. Early studies suggested better functional outcomes with off-clamp PN but longer operative time and greater blood loss [6,7]. More recent meta-analyses have provided inconsistent conclusions regarding the impact of clamping technique on renal function, perioperative morbidity, and oncological safety [8,9,10]

Given the growing body of literature and variability in surgical techniques, we performed an updated systematic review, meta-analysis, and meta-regression to provide comprehensive evidence comparing off-clamp and on-clamp PN across functional, and perioperative outcomes, as well as surgical margin status.

## 2. Materials and Methods

### 2.1. Search Strategy

This review was designed to systematically evaluate studies comparing off-clamp and on-clamp PN. The review protocol was preregistered in the PROSPERO database (registration number CRD42024614025). The Preferred Reporting Items for Systematic Reviews and Meta-Analyses (PRISMA) 2020 statement and the Cochrane Handbook for Systematic Reviews of Interventions (version 6.5, 2024) were followed for the study methodology [11,12]. The PRISMA 2020 checklist is provided in Supplementary Materials in Table S2, a template list of full-text exclusions with reasons in Table S3, and a GRADE summary of findings in Table S4.

A systematic literature search was conducted in the PubMed, Embase, Cochrane Library, Web of Science, and Scopus databases in 13 November 2024. The following search strategy was applied across all databases: (partial nephrectomy OR NSS OR nephron sparing surgery) AND (ischemia OR ischemic OR ischemic time OR infarction) AND (function OR insufficiency OR chronic kidney disease OR acute kidney disease OR AKI OR CKD) AND (renal cell cancer OR RCC OR kidney cancer). In PubMed, Medical Subject Headings (MeSH), and in Embase, exploded Emtree terms were used to construct the search queries. Two authors (Z.S. and M.J.) independently screened titles and abstracts after duplicate removal, with disagreements resolved by consulting a senior author (P.D.).

### 2.2. Study Selection

We developed the search strategy using the PICOS (Population, Intervention, Comparator, Outcome, Study design) framework. We included studies of patients diagnosed with renal cell carcinoma (RCC) at clinical stage cT1–2 (Population). Studies reporting incidental pathological upstaging to pT3a were also included, provided the primary surgical intent was nephron-sparing surgery for clinical T1–T2 disease. We included patients treated with PN (robotic [RAPN], laparoscopic [LPN] or open [OPN] approach), using the on-clamp technique (Intervention), and were compared with patients treated with the off-clamp PN (Comparison). The on-clamp group primarily included studies utilizing main renal artery or full hilar clamping, including those with early unclamping maneuvers where reported. The off-clamp group consisted of procedures performed without any intentional global renal ischemia. We acknowledge that variations in surgeon-specific techniques and the evolution of robotic platforms over the study period contribute to the observed clinical heterogeneity. We analyzed the estimated glomerular filtration rate (eGFR) differences and percentage eGFR changes, both at last follow-up, as primary outcomes, and operative time, estimated blood loss (EBL), blood transfusion, complication, and positive surgical margin (PSM) rates as secondary endpoints (Outcome). Eligible study designs included comparative studies, both RCTs and observational studies (retrospective or prospective, with or without patient matching), that directly evaluated off-clamp versus on-clamp PN (Study design). To be considered, studies had to report at least one of the prespecified outcomes. Only studies published in English enrolling adult patients (≥18 years old) were included.

We excluded reviews, meta-analyses, editorials, commentaries, letters, meeting abstracts, case reports or case series (<30 patients), studies with overlapping population or articles not written in English. Studies limited to nononcologic renal lesions, studies assessing cold ischemia, or reports in which the ischemia technique was not clearly specified, were excluded as well. Finally, we excluded studies that exclusively evaluated patients with a solitary kidney, horseshoe kidney, or transplanted kidney, as well as studies focusing solely on multiple renal masses.

### 2.3. Data Extraction

Data on studies, patients, and management characteristics were extracted independently by two authors (Z.S. and M.J.). For our study endpoints, we retrieved mean differences (MDs) for continuous variables and odds ratios (ORs) for categorical outcomes, both with corresponding 95% confidence intervals (CIs). Absolute number of events (i.e., blood transfusions, complications, and PSM rates) were retrieved as well. When standard deviations (SDs) were unavailable, they were estimated from interquartile ranges (IQRs) or standard errors (SEs) as recommended in the Cochrane Handbook for Systematic Reviews of Interventions (version 6.5, 2024). In studies reporting results as median (range) or median (IQR), data were converted to means and SDs to enable consistent quantitative pooling [13]. The transformations were based on the median, minimum, maximum, and sample size for ranges, and on Q1, Q3, and sample size for IQR data.

### 2.4. Risk of Bias and Quality Assessment

Two authors independently assessed the risk of bias (RoB) with the Cochrane Collaboration’s RoB tool for RCTs [12] and Newcastle-Ottawa Scale (NOS) [14] for observational studies. We generated funnel plots and performed Egger’s regression tests to statistically evaluate funnel plot asymmetry. A statistically significant result (*p* < 0.05) would suggest the presence of small-study effects or publication bias, whereas a non-significant result would indicate that the findings are robust.

### 2.5. Statistical Analysis

We performed meta-analyses and generated forest plots to assess pooled MDs and ORs. Data were synthesized using random-effects models due to anticipated significant between-study heterogeneity, which was evaluated using the Cochran Q (χ^2^) test and the I^2^ statistic. The between-study variance (τ^2^) was estimated using the DerSimonian-Laird (DL) method. Confidence intervals for the pooled effect size were calculated using the standard Wald-type method.

In cases of statistically significant heterogeneity, we performed univariable meta-regressions comprising ten different moderators to explore potential sources of heterogeneity. The moderators included: surgical technique, time of renal function assessment, study design, patient matching, sample size, RENAL nephrometry score, preoperative eGFR, operative time, and warm ischemia time. Additionally, we performed sensitivity analyses to reveal if particular studies dominated the results. All statistical analyses were conducted using either RStudio, version 2026.01.0+392 (R version 4.5.1), or Review Manager (RevMan), version 5.4 (Cochrane Collaboration). Statistical significance was set at *p* < 0.05.

## 3. Results

### 3.1. Study Selection and Characteristics

**Figure 1:**
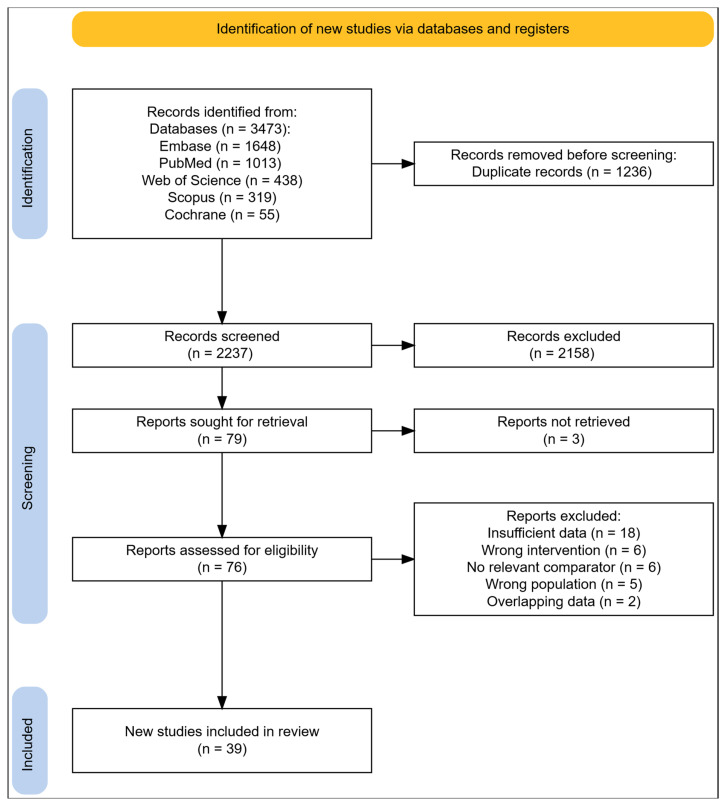
Preferred Reporting Items for Systematic Reviews and Meta-Analyses (PRISMA) flow chart.

The PRISMA flowchart is presented in Figure 1. A total of 3473 records were identified via databases. After removing 1236 duplicates, 2237 titles and abstracts were screened. Ultimately, 39 studies met the inclusion criteria [4,6,7,15,16,17,18,19,20,21,22,23,24,25,26,27,28,29,30,31,32,33,34,35,36,37,38,39,40,41,42,43,44,45,46,47,48,49,50]. The characteristics of included studies are presented in detail in Table 1.

In total, 10,154 patients were included. Among 39 included articles, there were 4 RCTs and 35 observational studies. Most cohorts were RAPN- or LPN-dominant, with OPN included less frequently. The primary functional endpoints were the absolute and relative changes in renal function (ΔeGFR and %ΔeGFR) assessed at the latest available follow-up. A total of 14 studies assessing eGFR differences and 11 studies reporting percentage change in eGFR were included. Most observational studies used matched or adjusted designs (e.g., propensity score or multivariable control); the most common matching variables were age, tumour size, RENAL nephrometry score, baseline eGFR/serum creatinine, and the American Society of Anesthesiologists (ASA) physical status. The three RCTs compared off-clamp vs. on-clamp PN within standardized perioperative protocols (two RAPN-based trials and one mixed/minimally invasive trial), reporting renal functional change and key perioperative endpoints. Geographically, the evidence spans North America, Europe, and Asia, with multi-centre contributions in several larger series. Follow-up durations varied across studies (short-term postoperative through ≥12 months). In 12 studies, medians with IQRs or ranges were converted to means and SDs for the relevant variables.

#### RoB, Quality Assessment, and Sensitivity Analysis

The summary of the RoB and quality assessment is presented in Supplementary Figures S1 and S2. Funnel plots assessing the risk of bias visually are presented in Supplementary Figures S3–S9.

The highest methodological reliability (lowest overall RoB) was observed in the CLOCK and CLOCK II trials, as well as in the study by Anderson et al., although all surgical RCTs inherently suffer from limitations in Domain 2 (deviations from the intended interventions) due to the impossibility of blinding the operating surgeons and the allowance for intraoperative strategy adjustments [4,16,19]. Consequently, the overall judgment for the three contemporary RCTs was rated as ‘some concerns’, primarily driven by Domain 2 issues rather than randomization or outcome measurement bias. The study by Voylenko et al. was rated as having a high RoB, mainly due to methodological and reporting deficiencies, including insufficient information on randomization procedures and incomplete outcome data [48]. For the observational studies, NOS scores ranged from 5 to 9 points (out of 9). The majority (over 75%) achieved moderate-to-high quality ratings (≥6 points). Most studies scored strongly in the selection and outcome domains, reflecting adequate cohort definition and reliable outcome assessment. However, comparability across groups was often limited due to incomplete adjustment for potential confounders in non-randomized designs. Sensitivity analysis revealed that no single study dominated the results in cases of all performed meta-analyses.

Overall, the methodological quality of the included studies was acceptable. The available evidence can therefore be considered moderately robust, with the main potential bias stemming from residual confounding in observational analyses rather than from randomization or reporting issues.

### 3.2. Meta-Analyses

#### 3.2.1. Estimated Glomerular Filtration Rate

**Figure 2:**
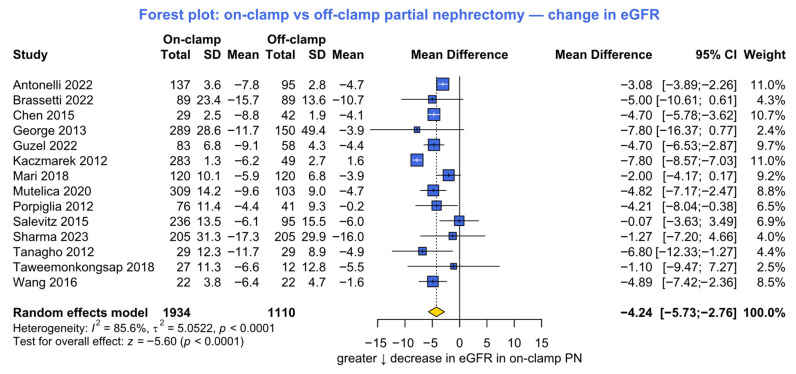
Forest plot presenting the primary analysis of absolute decline in estimated glomerular filtration rate (eGFR). SD = standard deviation; CI = confidence interval; PN = partial nephrectomy [6,7,16,21,23,26,27,33,34,36,40,41,44,49].

Change in eGFR was reported in 14 studies including 3044 patients (1934 on-clamp vs. 1110 off-clamp). The pooled analysis revealed a significant advantage for the off-clamp technique, with a smaller decline in renal function compared to on-clamp PN (MD −4.2, 95% CI −5.7 to −2.8, *p* < 0.001; Figure 2). The study heterogeneity was statistically significant (*p* < 0.001).

#### 3.2.2. Percentage Change in Estimated Glomerular Filtration Rate

**Figure 3:**
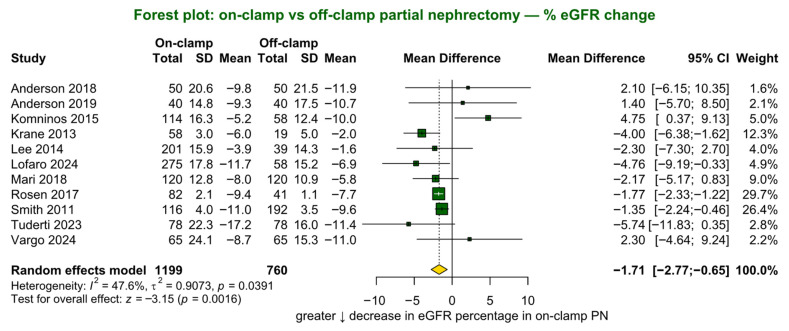
Forest plot presenting the primary analysis of percentage decline in estimated glomerular filtration rate (eGFR). SD = standard deviation; CI = confidence interval [4,15,28,30,31,32,33,39,43,45,46].

Percentage change in eGFR was reported in 11 studies (*n* = 1959; 1199 on-clamp vs. 760 off-clamp). The pooled analysis showed a statistically significant benefit for the off-clamp approach, with a smaller decline in renal function (MD −1.7%, 95% CI −2.8% to −0.7%, *p* = 0.002; Figure 3). The study heterogeneity was statistically significant (*p* = 0.04).

#### 3.2.3. Operative Time

Twenty-eight studies (*n* = 5830; 3671 on-clamp vs. 2159 off-clamp) reported operative time. The pooled analysis showed no significant difference between the two approaches (MD 17 min, 95% CI −1.5 to 36, *p* = 0.07; Supplementary Figure S10). Heterogeneity across studies was statistically significant (*p* < 0.001), indicating variability in surgical practice, patient selection, and reporting methods.

#### 3.2.4. Estimated Blood Loss

**Figure 4:**
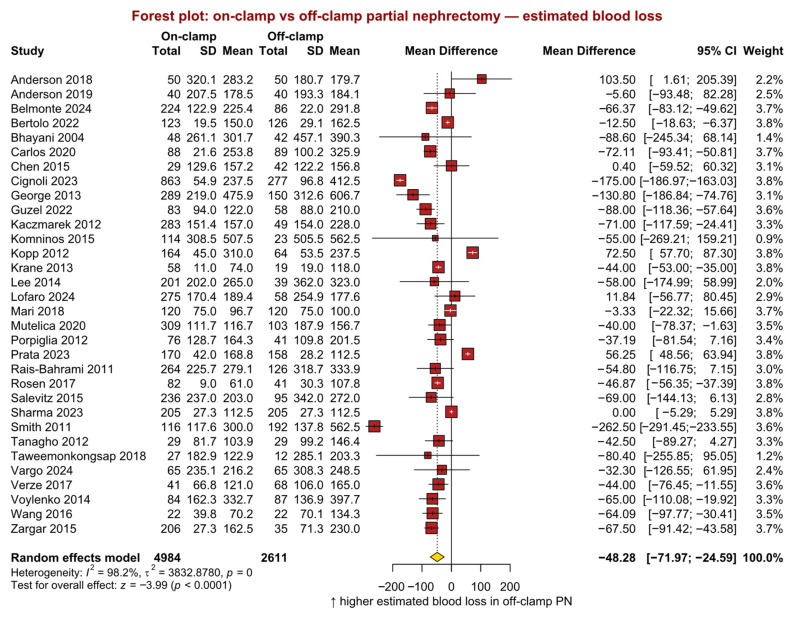
Forest plot presenting the primary analysis of estimated blood loss between on-clamp and off-clamp partial nephrectomy. SD = standard deviation; CI = confidence interval [4,6,7,15,17,19,20,22,23,24,26,27,28,29,30,31,32,33,34,36,37,38,39,40,41,43,44,46,47,48,49,50].

Thirty-two studies (*n* = 7595; 4984 on-clamp vs. 2611 off-clamp) reported EBL. The pooled analysis demonstrated a statistically significant difference favouring the on-clamp technique (MD −48 mL, 95% CI −72 to −25, *p* < 0.001; Figure 4). Study heterogeneity was statistically significant (*p* < 0.001).

#### 3.2.5. Blood Transfusion Rate

Twenty-two studies (*n* = 6468; 4306 on-clamp vs. 2162 off-clamp) reported blood transfusion rates. The pooled analysis showed no statistically significant difference between groups (OR 0.7, 95% CI 0.5 to 1.0, *p* = 0.08, Supplementary Figure S11). Study heterogeneity was statistically significant (*p* = 0.003).

#### 3.2.6. Complication Rate

**Figure 5:**
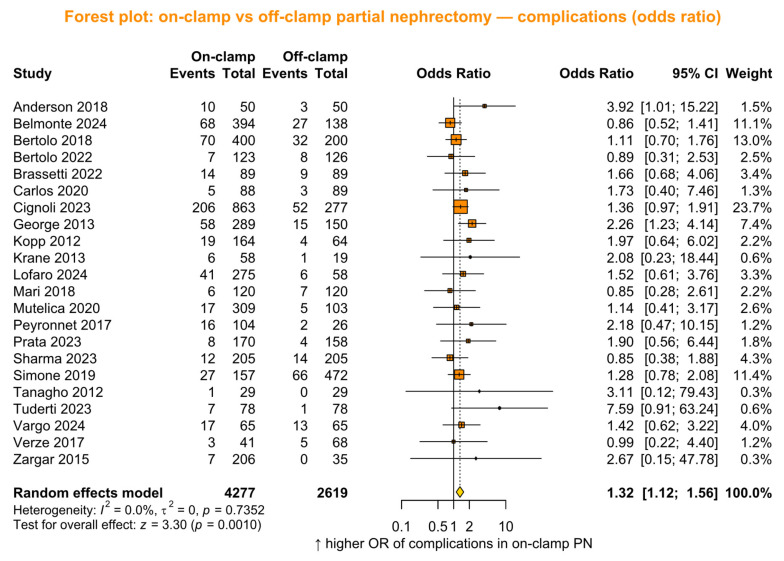
Forest plot presenting the primary analysis of complications odds ratio (OR) between on-clamp and off-clamp partial nephrectomy (PN). CI = confidence interval [6,7,15,17,18,19,21,22,24,29,30,32,33,34,35,37,41,42,45,46,47,50].

Complication rates were reported in 22 studies (*n* = 6896; 4277 on-clamp vs. 2619 off-clamp). The pooled analysis showed a significantly higher OR of complications in the on-clamp group (OR 1.32, 95% CI 1.12 to 1.56, *p* = 0.003; Figure 5). Due to the homogeneity of the data (*p* = 0.73), meta-regression was not performed.

#### 3.2.7. Positive Surgical Margin Rates

**Figure 6:**
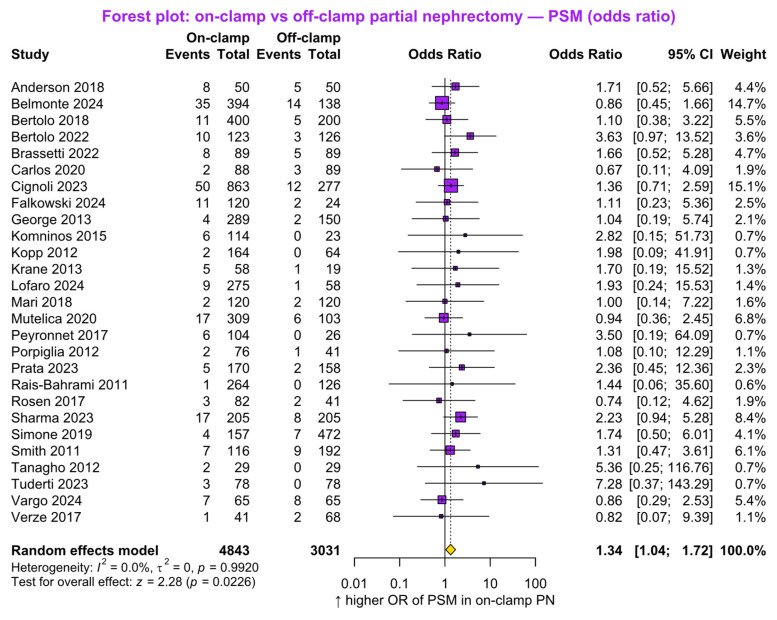
Forest plot presenting the odds ratio (OR) of positive surgical margin (PSM) between on-clamp and off-clamp partial nephrectomy (PN). CI = confidence interval [6,7,15,17,18,19,22,24,25,28,29,30,32,33,34,35,36,37,38,39,41,42,43,45,46,47].

Twenty-seven studies (*n* = 7874; 4843 on-clamp vs. 3031 off-clamp) reported PSM rates. The pooled analysis demonstrated a significantly higher OR of PSM in the on-clamp group (OR 1.34, 95% CI 1.04 to 1.72, *p* = 0.02; Figure 6). No statistically significant study heterogeneity was detected (*p* = 0.99).

### 3.3. Meta-Regression Analyses

The results of the meta-regression analyses are presented in Supplementary Table S1. Univariable meta-regression analyses were conducted for outcomes that demonstrated both statistical significance and between-study heterogeneity (*p* < 0.05), namely eGFR, percentage change in eGFR, and EBL. In the eGFR analysis, the RENAL nephrometry score was the only moderator significantly associated with effect size. No statistically significant moderators were identified in the meta-regression of percentage eGFR change. For EBL, both the RENAL nephrometry score and sample size emerged as significant moderators. All remaining covariates did not reach statistical significance. We did not perform the multivariable meta-regression due to the limited number of eligible studies relative to the number of moderators and the predominance of non-significant variables, which would have increased the risk of unstable estimates.

## 4. Discussion

We conducted an updated systematic review, meta-analysis, and meta-regression of comparative studies that analysed the differences between on-clamp and off-clamp PN in all surgical modalities (robot-assisted, laparoscopic, and open) in renal function, perioperative outcomes, and surgical margin status. Warm ischemia time remains a critical determinant of postoperative renal function after PN, yet its exact clinical impact continues to be debated. Our analysis demonstrated a mean difference of 4 mL/min/1.73 m^2^ in favour of the off-clamp approach, corroborating the hypothesis that eliminating ischemia mitigates parenchymal injury. Importantly, the benefit persisted across surgical modalities and at both short- and long-term follow-up in meta-regressions, suggesting a durable functional effect. Nevertheless, the magnitude of benefit may have limited clinical relevance in patients with normal baseline renal function and contralateral kidney compensation, as such a difference is unlikely to trigger a shift in CKD staging. It becomes more pertinent in patients with solitary kidneys, baseline chronic kidney disease, or complex tumours requiring extensive parenchymal excision, where preserving even a small increment of glomerular filtration rate can prevent progression to a more advanced CKD category and its associated cardiovascular risks. Thus, while statistically significant, the real-world impact of warm ischemia avoidance should be interpreted in the context of individual renal reserve and clinically meaningful thresholds.

Our finding that off-clamp PN better preserves renal function is consistent with meta-analyses by Deng et al. [8] and Mina-Riascos et al. [9], both of which reported smaller eGFR declines in off-clamp cohorts. Similarly, Huang et al. [5] conducted the largest robotic-only meta-analysis to date (21 studies, 4493 patients), demonstrating a significantly smaller long-term %eGFR decrease (weighted MD −3.2, 95% CI −5.8 to −0.5) favouring off-clamp RAPN. Their findings support the concept of renal protection without ischemia. However, between-study heterogeneity was high and the difference largely disappeared after excluding one large study [16]. In contrast, meta-analyses by Cacciamani et al. [51] and Antonelli et al. [52] reported no clinically relevant eGFR differences in robotic or minimally invasive series with statistically-similar RENAL score, suggesting that when warm ischemia is brief and tumour complexity is lower, functional equivalence may be achievable. Likewise, the European Association of Urology–Young Academic Urologists (EAU–YAU) collaborative meta-analysis [10] reinforced this notion, finding no difference in percentage eGFR decline between on- and off-clamp techniques in matched robotic cohorts.

We confirmed higher estimated blood loss in off-clamp PN, consistent with previous investigations [5,52,53], where increased bleeding did not translate into higher transfusion rates. While this difference reached statistical significance, the absolute mean difference of ~48 mL is clinically modest and likely falls within the margin of error inherent in visual intraoperative estimation. It is important to acknowledge that EBL remains a subjective measure with potential for significant variability across different surgical teams and institutions. Consequently, for the vast majority of patients, this slight increase in EBL associated with the off-clamp technique is unlikely to be clinically relevant in terms of transfusion requirements or overall postoperative recovery. Huang et al. [5] also found shorter operative time and lower overall complication rates in the off-clamp group, though case selection bias (smaller tumours, lower RENAL score) was noted. However, our analysis showed no significant difference between the two approaches. Furthermore, we observed higher risk of complications with on-clamp PN, which aligns with one of the previous quantitative analyses [52].

Our pooled data identified a higher risk of PSM in on-clamp PN, consistent with the findings reported by Antonelli et al. [52], Shrivastava et al. [10], and Huang et al. [5], who reported either no difference or slightly lower PSM with off-clamp RAPN. Cacciamani et al. [51] found no statistically significant differences in PSM across open, laparoscopic, and robotic cohorts, suggesting that oncologic outcomes are more strongly influenced by tumour complexity and resection strategy (standard PN vs. enucleation) than by the clamping technique itself. Conversely, our study shows that the PSM rate is higher in on-clamp cohorts. This finding, however, should be interpreted with caution. Although vascular clamping is generally assumed to improve surgical field visibility, the observed difference is most likely influenced by selection bias, as off-clamp techniques are more frequently applied in patients with less complex tumors, including smaller and more exophytic lesions, which are inherently associated with a lower risk of incomplete resection. In addition, the absence of time pressure related to warm ischemia may allow for a more controlled tumor excision. Furthermore, surgeon experience may represent an additional confounding factor, as off-clamp procedures are often performed in high-volume centers. Although our meta-analysis did not reveal statistically significant between-study heterogeneity, this divergence may still reflect residual confounding and biases associated with predominantly retrospective, single-centre data rather than a true oncological advantage of the off-clamp approach.

Relative to previous, platform-restricted analyses, our meta-analysis integrates a broader evidence base encompassing multiple surgical approaches, and both randomized controlled and observational data. Our results converge with Huang et al. [5], Deng et al. [8], and Mina-Riascos et al. [9] in demonstrating a measurable renal benefit of ischemia avoidance, while diverging from robotic-only syntheses (Cacciamani et al. [51], Antonelli et al. [52], Shrivastava et al. [10]) that suggest functional equivalence under optimized robotic conditions. Compared with the recent large-scale update by Serag et al. [54], our findings do not support on-clamp superiority in renal function but confirm a trade-off between functional preservation, and increased bleeding risk.

In unselected, multi-platform clinical practice, off-clamp PN is associated with superior preservation of eGFR and less positive surgical margins during tumour excision, at the cost of a modest increase in intraoperative bleeding. Meta-regression analyses identified the RENAL nephrometry score as the only consistent moderator of effect size in two of the three evaluated outcomes. Accordingly, the choice of clamping strategy should be guided by careful patient selection, primarily driven by tumour size and complexity, as well as surgeon experience. For complex or hilar tumours requiring optimal surgical exposure, a short and controlled period of warm ischemia may represent a pragmatic compromise that does not adversely affect long-term renal function, in line with evidence from robotic-assisted surgery series.

Despite the use of meta-regression, which identified the RENAL nephrometry score as a significant contributor to heterogeneity in both functional (eGFR) and perioperative (EBL) outcomes, residual heterogeneity remains and likely reflects additional unmeasured factors, including variability in surgical approach, ischemia management, and surgeon experience. Importantly, the certainty of evidence for most outcomes was rated as low to very low according to GRADE, primarily due to the predominance of observational studies and the inherent risk of selection bias. Therefore, the observed differences between off-clamp and on-clamp techniques should be interpreted with caution and considered hypothesis-generating rather than definitive. Further high-quality randomized controlled trials are needed to provide more robust evidence.

The incremental value of our study lies in its scale and methodological breadth. By pooling data from 10,154 patients, we provide the highest statistical power of any meta-analysis on this topic to date. Moreover, by including all surgical modalities (RAPN, LPN, and OPN), our findings are applicable across diverse clinical settings. Crucially, our use of the RENAL nephrometry score as a moderator in meta-regression models offers a more sophisticated understanding of the on-clamp vs. off-clamp debate, moving beyond generalized comparisons to a more individualized, complexity-based framework.

Our study has several important limitations. First, owing to the limited number of available RCTs, the meta-analysis relies predominantly on observational data, which are inherently associated with increased RoB. Second, substantial between-study heterogeneity was observed, likely reflecting retrospective study designs, inconsistent reporting of ischemia duration and tumour complexity, and limited availability of long-term follow-up data. Third, residual confounding related to surgeon preference, surgical expertise, and perioperative management strategies cannot be fully excluded. Fourth, although meta-regression analyses suggested potential contributors to heterogeneity, most notably tumour complexity as assessed by the RENAL nephrometry score, no single variable could be identified as a definitive source of heterogeneity. Finally, we did not specifically analyse patients with chronic kidney disease or a solitary kidney, populations in whom renal function preservation is of greater clinical importance than in the general PN population.

## 5. Conclusions

Taken together, our findings suggest that avoidance of renal hilar clamping during PN is associated with improved preservation of renal function and lower risks of PSMs and postoperative complications, at the cost of increased intraoperative bleeding. These data underscore the importance of individualised surgical planning that accounts for patient characteristics, tumour anatomy, and surgical expertise, and indicate that off-clamp techniques may confer an overall benefit in carefully selected patients. Nevertheless, well-designed RCTs and large multicenter prospective registries remain necessary to better define the long-term functional and oncological consequences of ischemia avoidance.

## Figures and Tables

**Table 1:** Characteristics of the 39 included studies.

First Author (Year)	Country	Study Setting	No. of Patients Overall	No. of Patients (On-Clamp/Off-Clamp)	T Stage	Surgical Technique	Renal Function Measurement Method	Time of Renal Function Assessment
Anderson (2018) [15]	USA	RS, PSM	100	50/50	cT1	RAPN	%ΔeGFR	<12 m
Anderson (2019) [4]	USA	RCT	80	40/40	cT1	RAPN	%ΔeGFR	2.3–4.5 m
Antonelli (2022) [16]	Italy	RCT	324	160/164 (PPA 137/95)	cT1	RAPN	ΔeGFR	6 m
Belmonte (2024) [17]	Belgium	RS, IPTW	532	394/138 (IPTW 224/86)	cT1–2	RAPN	eGFR, sCr	1 m
Bertolo (2018) [18]	USA/Italy	RS, PSM	600	400/200	pT1–T3a	RAPN	eGFR	NA
Bertolo (2022) [19]	Italy	RCT	249	123/126	cT1	LPN	eGFR	5 d
Bhayani (2004) [20]	USA	RS	90	48/42	cT1	LPN	%ΔsCr	6 m
Brassetti (2022) [21]	Multi-center	RS	316	211/105 (PSM 89/89)	cT2	RAPN	ΔeGFR	<5 d
Carlos (2020) [22]	Brazil	RS	177	88/89	pT1a–T2	LPN	eGFR, sCr	<1 m
Chen (2015) [23]	China	PNRS	71 (subgroups)	29/42	cT1	LPN	ΔeGFR	1 m
Cignoli (2023) [24]	Italy	RS	1140	863/277	cT1–T2	OPN, LPN, RAPN	eGFR	6 m
Falkowski (2024) [25]	Poland	RS	144	120/24	cT1	LPN	eGFR	1 d
George (2013) [7]	USA	RS	439	289/150	NA	LPN	ΔeGFR	6 m
Güzel (2022) [26]	Turkey	RS	141	83/58	NA	R-LPN	ΔeGFR	50.7–51.5 m
Kaczmarek (2012) [27]	USA	RS, PSM	886 (PSM 332)	820/66 (PSM 283/49)	NA	RAPN	ΔeGFR	6 m
Komninos (2015) [28]	Korea	RS	137	114/23	pT1–T3a	RAPN	%ΔeGFR	12 m
Kopp (2012) [29]	USA	RS	228	164/64	cT1	OPN	NA	24–53 m
Krane (2013) [30]	USA	PNRS	77	58/19	NA	RAPN	%ΔeGFR, %ΔsCr	4 m
Lee (2014) [31]	Korea	RS	240	201/39	cT1a	OPN	%ΔeGFR	12 m
Lofaro (2024) [32]	Italy	RS, EBW	333	275/58	cT1–T2	RAPN, LPN	%ΔeGFR	NA
Mari (2018) [33]	Italy	RS, PSM	240	120/120	cT1	RAPN	ΔeGFR, %ΔeGFR	6 m
Mutelica (2020) [34]	France	RS, PSM	940 (PSM 412)	837/103 (PSM 309/103)	cT1a	RAPN	ΔeGFR	6 m
Peyronnet (2017) [35]	France	RS, PSM	130	104/26	cT1	RAPN	%ΔeGFR	6 m
Porpiglia (2012) [36]	Italy	PNRS	117	76/41	cT1	LPN	ΔeGFR	<6 d
Prata (2023) [37]	Italy	RS	328	170/158	pT1–T3a	RAPN	eGFR	14–18 m
Rais-Bahrami (2011) [38]	USA	RS	390	264/126	cT1–T2	LPN	%ΔsCr	6 m
Rosen (2017) [39]	USA	RS, PSM	351 (PSM 123)	294/57 (PSM 82/41)	cT1a	RAPN	%ΔeGFR	9–14 m
Salevitz (2015) [40]	USA	RS	331	236/95	NA	OPN, LPN, RAPN	ΔeGFR	<3 m
Sharma (2023) [41]	India	RS, PSM	2114 (PSM 410)	1904/210 (PSM 205/205)	cT1	RAPN	ΔeGFR	17–19 m
Simone (2019) [42]	Italy	RS, PSM	1073 (PSM 629)	588/485 (PSM 157/472)	cT1–T2	NA	eGFR	49.2–50.2 m
Smith (2011) [43]	USA	RS	308	116/192	pT1–3	OPN, LPN, RAPN	%ΔeGFR	12 m
Tanagho (2012) [6]	USA	RS, PSM	58	29/29	cT1	RAPN	ΔeGFR	<12 m
Taweemonkongsap (2018) [44]	Thailand	RS	77	27/12 (38 excluded due to selective clamping)	pT1	RAPN	ΔeGFR	19–26 m
Tuderti (2023) [45]	Italy	RS, PSM	354 (PSM 156)	142/212 (PSM 78/78)	cT1–T2	RAPN	%ΔeGFR	NA
Vargo (2024) [46]	USA	RS, PSM	225 (PSM 130)	147/78 (PSM 65/65)	cT1b–T2	RAPN	%ΔeGFR	12 m
Verze (2017) [47]	Italy	RS	109	41/68	cT1–T2	LPN	eGFR, sCr	6 m
Voylenko (2014) [48]	Ukraine	RCT	171	84/87	NA	NA	eGFR	12 m
Wang (2016) [49]	China	RS	44	22/22	cT1	R-LPN	ΔeGFR	7 d
Zargar (2015) [50]	USA	RS	241	206/35	cT1	RAPN	eGFR	12–24 m

RS = retrospective study; PNRS = prospective non-randomized study; RCT = randomized controlled trial; PSM = propensity score-matched analysis; PPA = per-protocol analyses; IPTW = inverse probability of treatment weighting model; EBW = energy balancing weights method; OPN = open partial nephrectomy; LPN = laparoscopic partial nephrectomy; R-LPN = retroperitoneal laparoscopic partial nephrectomy; RAPN = robotic partial nephrectomy; eGFR = estimated glomerular filtration rate; %ΔeGFR = percentage change in eGFR; ΔeGFR = change in eGFR; sCr = serum creatinine; %ΔsCr = percentage change in sCR; NA = data not available; d = days; m = months.

## Data Availability

The original contributions presented in this study are included in the article/Supplementary Materials. Further inquiries can be directed to the corresponding author.

## Supplementary Materials

The following supporting information can be downloaded at: https://www.mdpi.com/article/10.3390/jcm15072792/s1, Figure S1—Risk of bias assessment of randomized controlled trials according to the Cochrane RoB 2.0. Figure S2—Quality assessment of observational studies using the Newcastle–Ottawa Scale. Figure S3—Funnel plot evaluating potential publication bias in the meta-analysis on estimated glomerular filtration rate (eGFR) changes. Figure S4—Funnel plot evaluating potential publication bias in the meta-analysis on percentage changes in estimated glomerular filtration rate (eGFR). Figure S5—Funnel plot evaluating potential publication bias in the meta-analysis on operative time. Figure S6—Funnel plot evaluating potential publication bias in the meta-analysis on estimated blood loss (EBL). Figure S7—Funnel plot evaluating potential publication bias in the meta-analysis on blood transfusion rates. Figure S8—Funnel plot evaluating potential publication bias in the meta-analysis on complication rates. Figure S9—Funnel plot evaluating potential publication bias in the meta-analysis on positive surgical margin (PSM) rates. Figure S10—Forest plot presenting the analysis of the operative time between on-clamp and off-clamp partial nephrectomy. Figure S11—Forest plot presenting the analysis of the blood transfusion rates between on-clamp and off-clamp partial nephrectomy. Table S1—Summary of univariable meta-regressions for the association of the estimated glomerular filtration rate (eGFR) changes, % changes in eGFR, and estimated blood loss (EBL) in off-clamp versus on-clamp partial nephrectomy. Table S2—The PRISMA 2020 checklist. Table S3—Full-text articles excluded, with the main reason for exclusion. Table S4—GRADE Summary of Findings. Table S5—Database-specific search strings.

## Abbreviations

PN—partial nephrectomy, eGFR—estimated glomerular filtration rate, MD—mean difference, OR—odds ratio, CI—confidence interval, PSM—positive surgical margin, EBL—estimated blood loss.
