# Supplementary material for: Off-Clamp Versus On-Clamp Partial Nephrectomy: An Updated Systematic Review, Meta-Analysis and Meta-Regression
Source: J Clin Med. 2026 Apr 7;15(7):2792. doi: 10.3390/jcm15072792 (PMC13073402; doi:10.3390/jcm15072792)
Supplement: Supplementary file 1 [file jcm-15-02792-s001.zip › jcm-4192458-supplementary.pdf]

Off-Clamp Versus On-Clamp Partial Nephrectomy: An Updated Systematic Review, Meta-Analysis and Meta-Regression

Table of Content

Off-clamp Versus On-clamp Partial Nephrectomy: An Updated Systematic Review, Meta-Analysis and Meta-Regression 1

Table of Content 1

Supplementary Figures 2

Supplementary Figure S1 - Risk of bias assessment of randomized controlled trials according to the Cochrane RoB 2.0. 2

Supplementary Figure S2 - Quality assessment of observational studies using the Newcastle–Ottawa Scale. 3

Supplementary Figure S3 - Funnel plot evaluating potential publication bias in the meta-analysis on estimated glomerular filtration rate (eGFR) changes. 4

Supplementary Figure S4 - Funnel plot evaluating potential publication bias in the meta-analysis on percentage changes in estimated glomerular filtration rate (eGFR). 4

Supplementary Figure S5 - Funnel plot evaluating potential publication bias in the meta-analysis on operative time. 5

Supplementary Figure S6 - Funnel plot evaluating potential publication bias in the meta-analysis on estimated blood loss (EBL). 5

Supplementary Figure S7 - Funnel plot evaluating potential publication bias in the meta-analysis on blood transfusion rates. 6

Supplementary Figure S8 - Funnel plot evaluating potential publication bias in the meta-analysis on complication rates. 6

Supplementary Figure S9 - Funnel plot evaluating potential publication bias in the meta-analysis on positive surgical margin (PSM) rates. 7

Supplementary Figure S10 - Forest plot presenting the analysis of the operative time between on-clamp and off-clamp partial nephrectomy. 7

Supplementary Figure S11 - Forest plot presenting the analysis of the blood transfusion rates between on-clamp and off-clamp partial nephrectomy. 8

Supplementary Tables 9

Supplementary Table S1 - Summary of univariable meta-regressions for the association of the estimated glomerular filtration rate (eGFR) changes, % changes in eGFR, and estimated blood loss (EBL) in off-clamp versus on-clamp partial nephrectomy. 9

Supplementary Table S2 - The PRISMA 2020 checklist 10

Supplementary Table S3 - Full-text articles excluded, with the main reason for exclusion. 12
Supplementary Table S4 - GRADE Summary of Findings 14
Supplementary Table S5 - Database-specific search strings 15

Supplementary Figures

Supplementary Figure S1 - Risk of bias assessment of randomized controlled trials according to the Cochrane RoB 2.0.

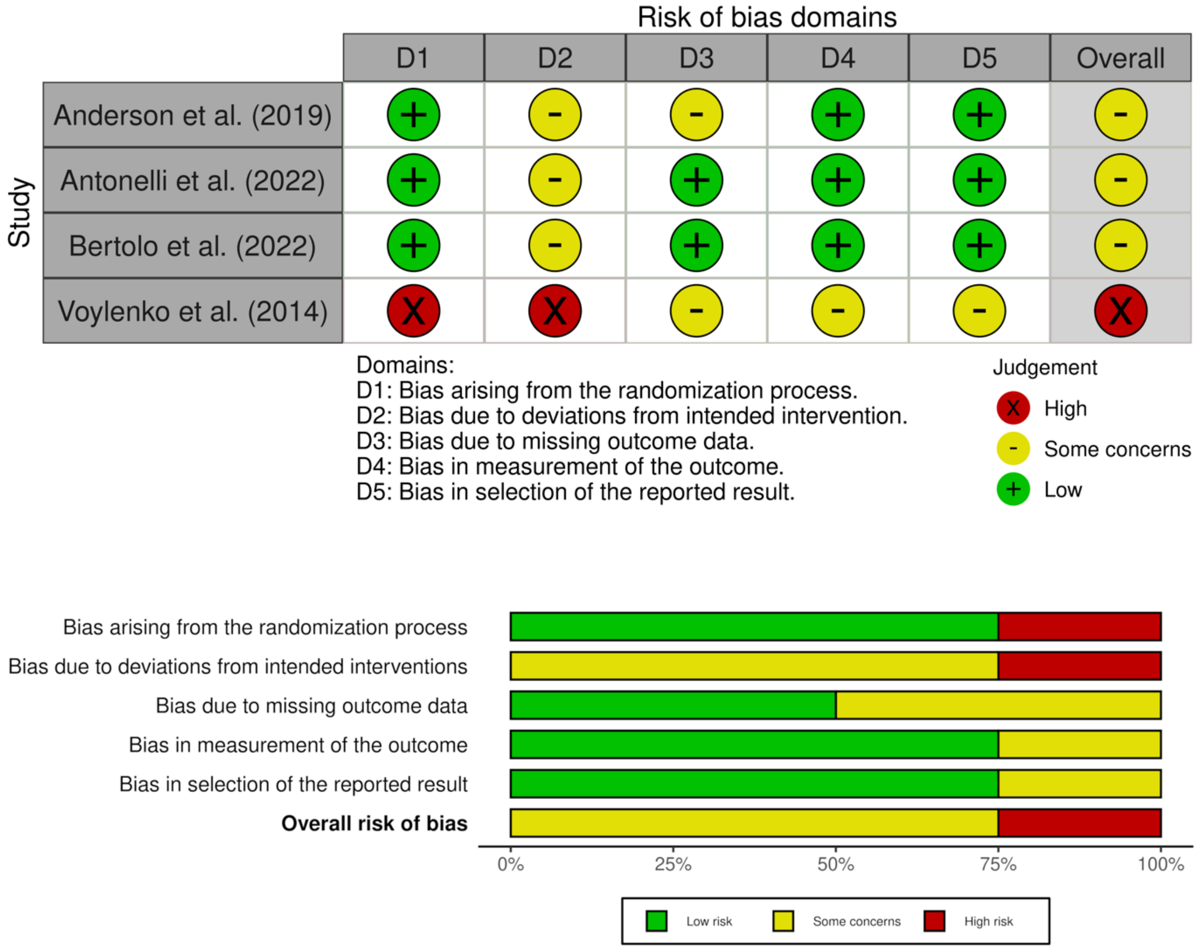

Supplementary Figure S2 - Quality assessment of observational studies using the Newcastle–Ottawa Scale.

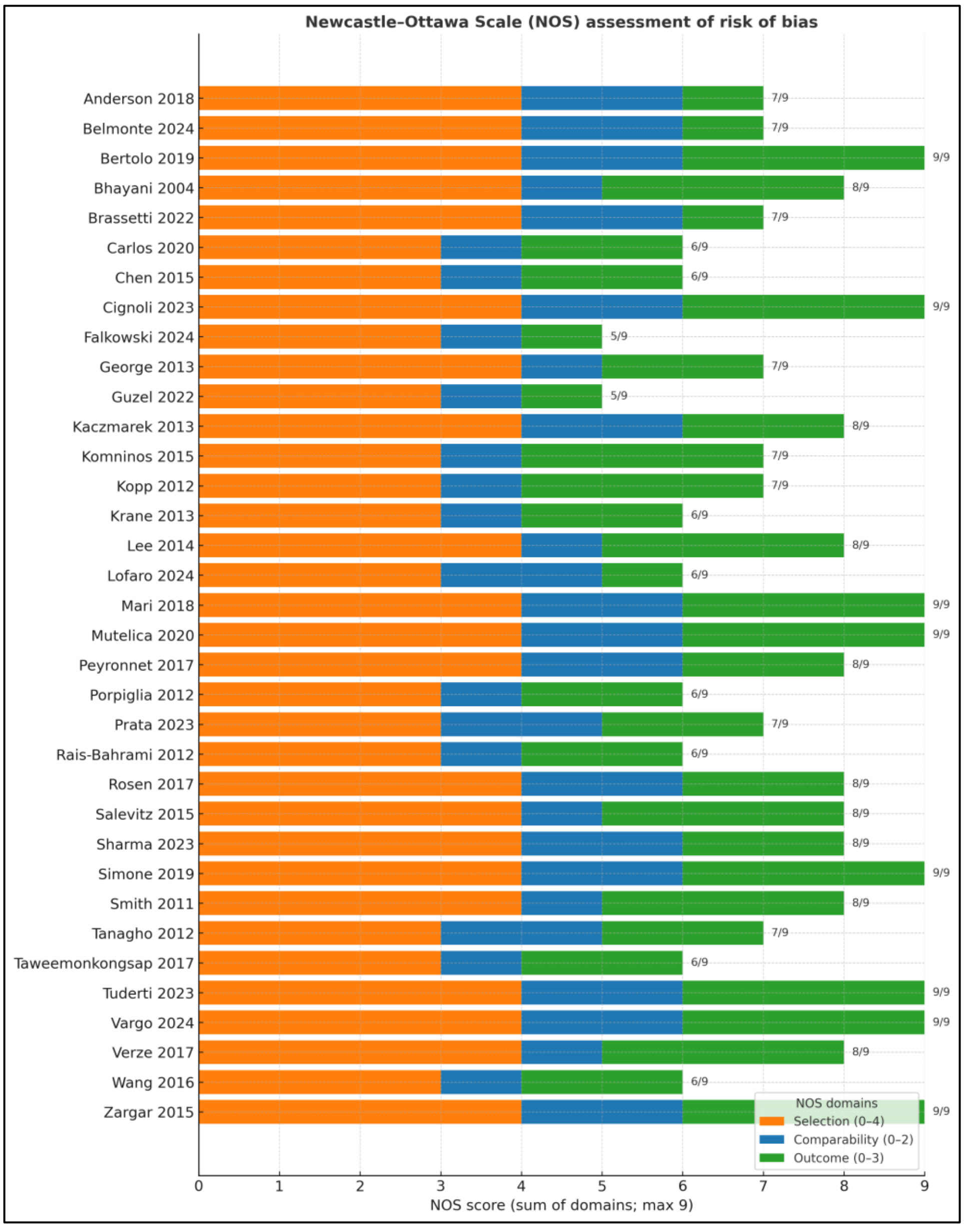

Supplementary Figure S3 - Funnel plot evaluating potential publication bias in the meta-analysis on estimated glomerular filtration rate (eGFR) changes.

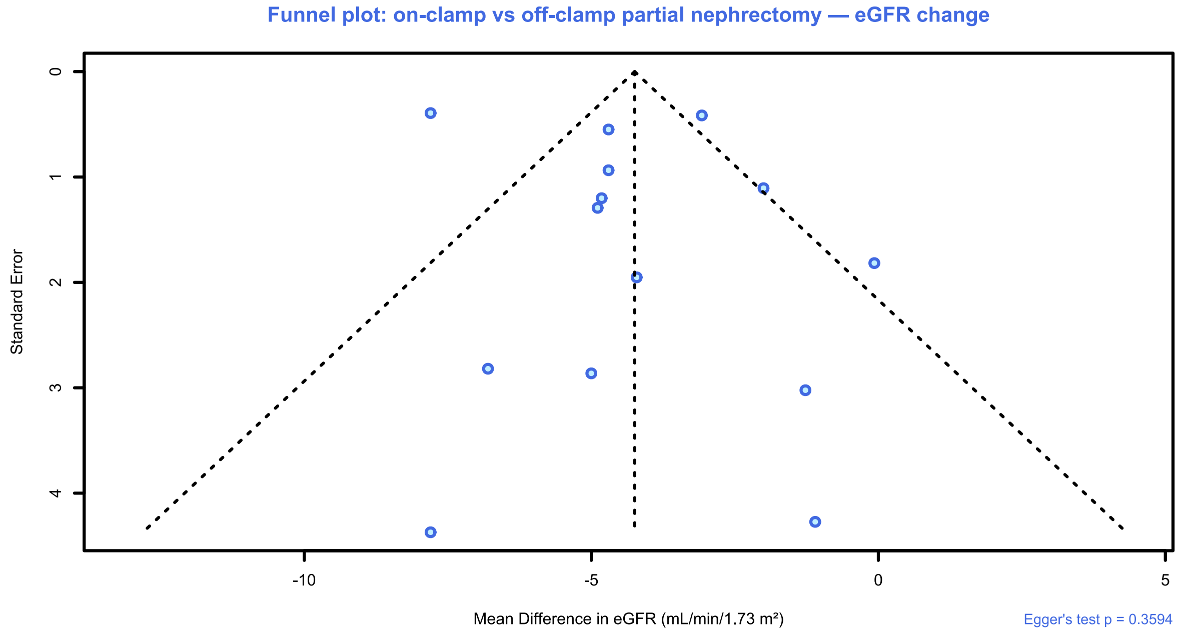

Supplementary Figure S4 - Funnel plot evaluating potential publication bias in the meta-analysis on percentage changes in estimated glomerular filtration rate (eGFR).

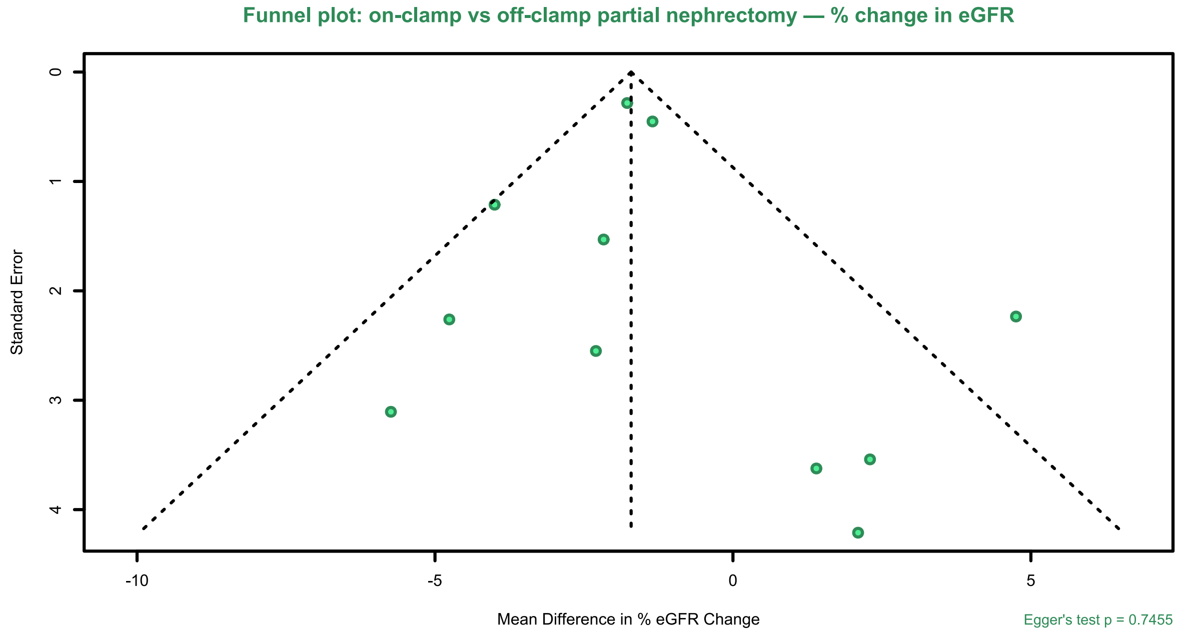

Supplementary Figure S5 - Funnel plot evaluating potential publication bias in the meta-analysis on operative time.

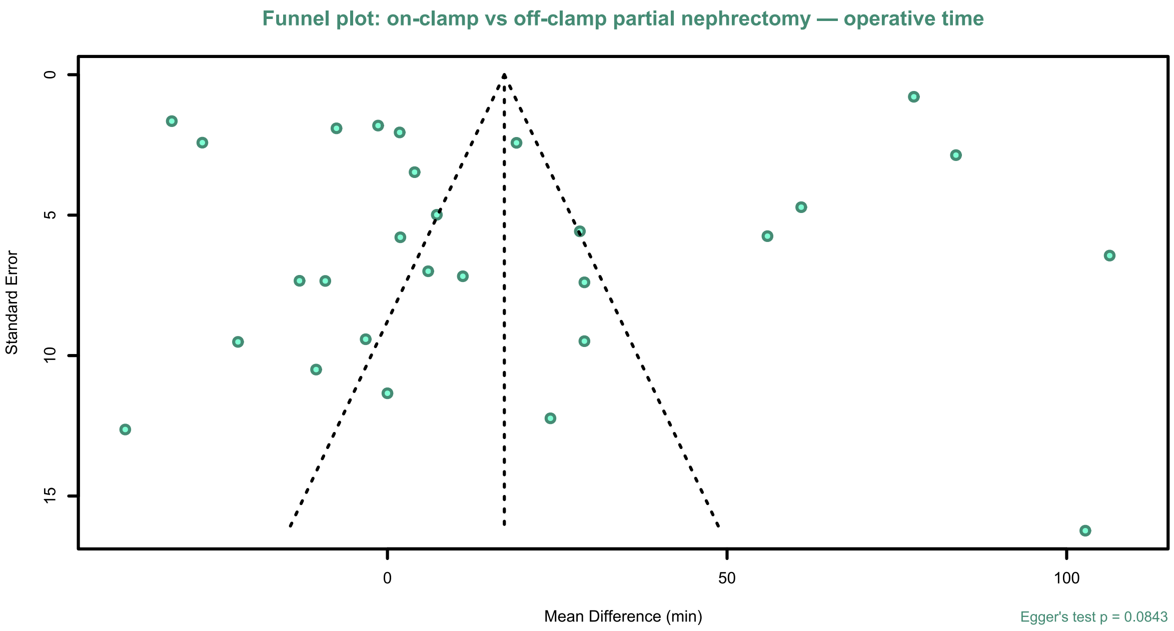

Supplementary Figure S6 - Funnel plot evaluating potential publication bias in the meta-analysis on estimated blood loss (EBL).

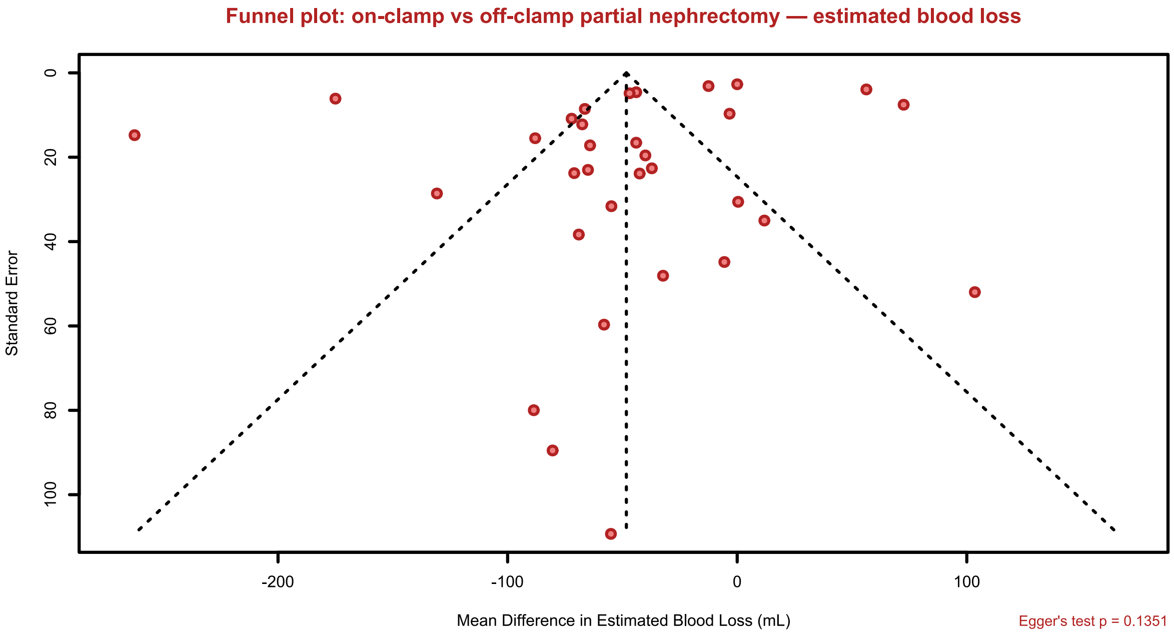

Supplementary Figure S7 - Funnel plot evaluating potential publication bias in the meta-analysis on blood transfusion rates.

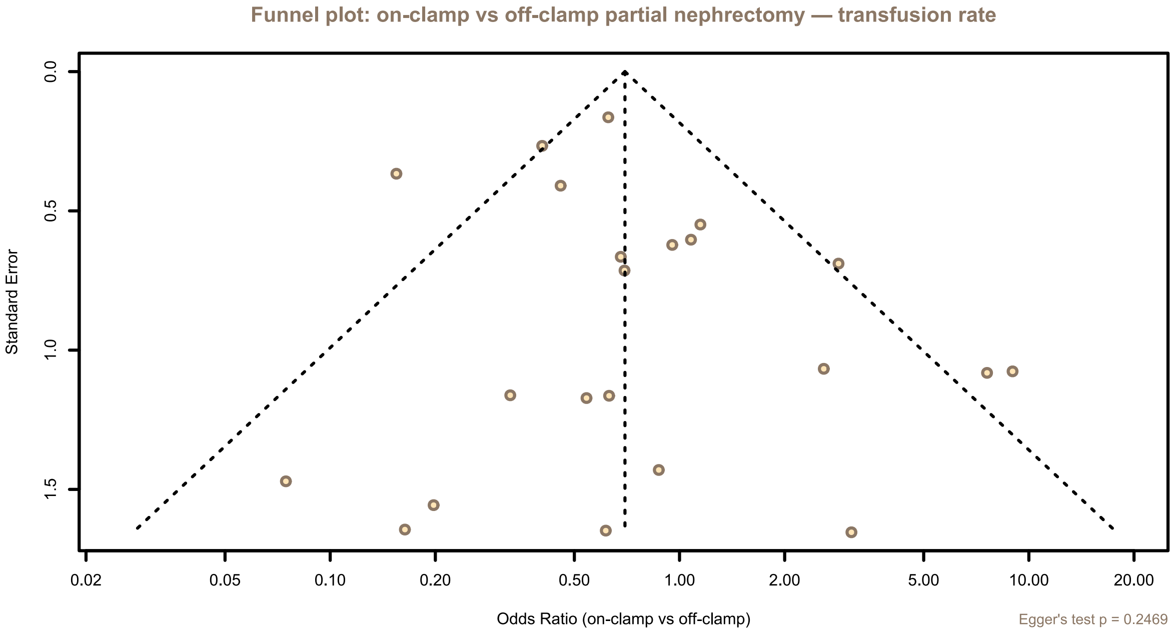

Supplementary Figure S8 - Funnel plot evaluating potential publication bias in the meta-analysis on complication rates.

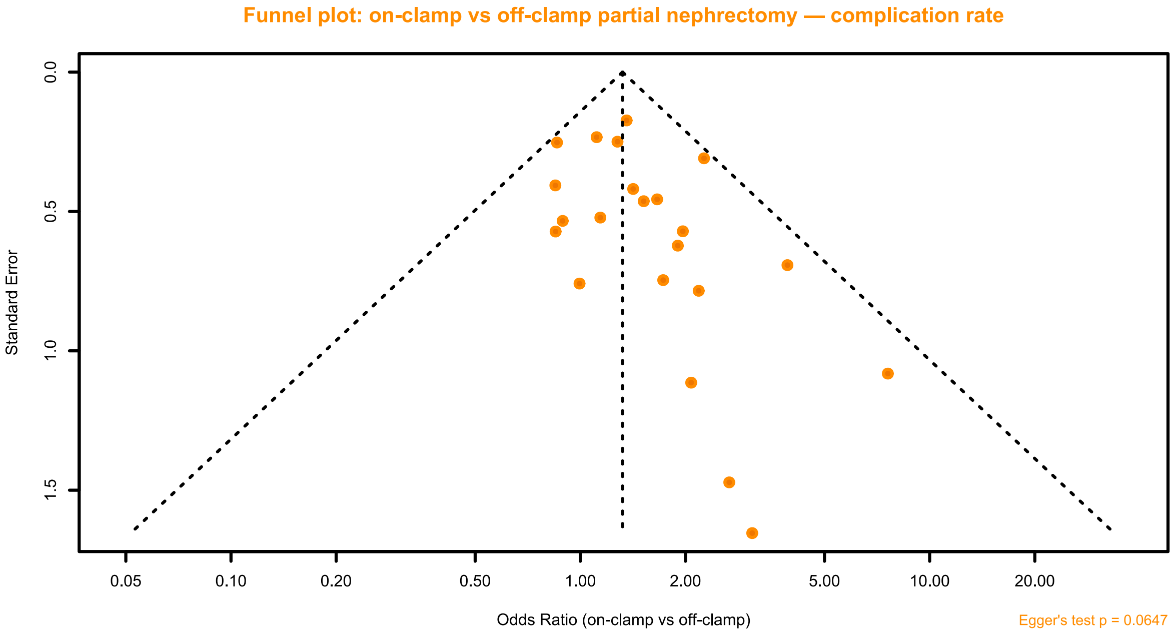

Supplementary Figure S9 - Funnel plot evaluating potential publication bias in the meta-analysis on positive surgical margin (PSM) rates.

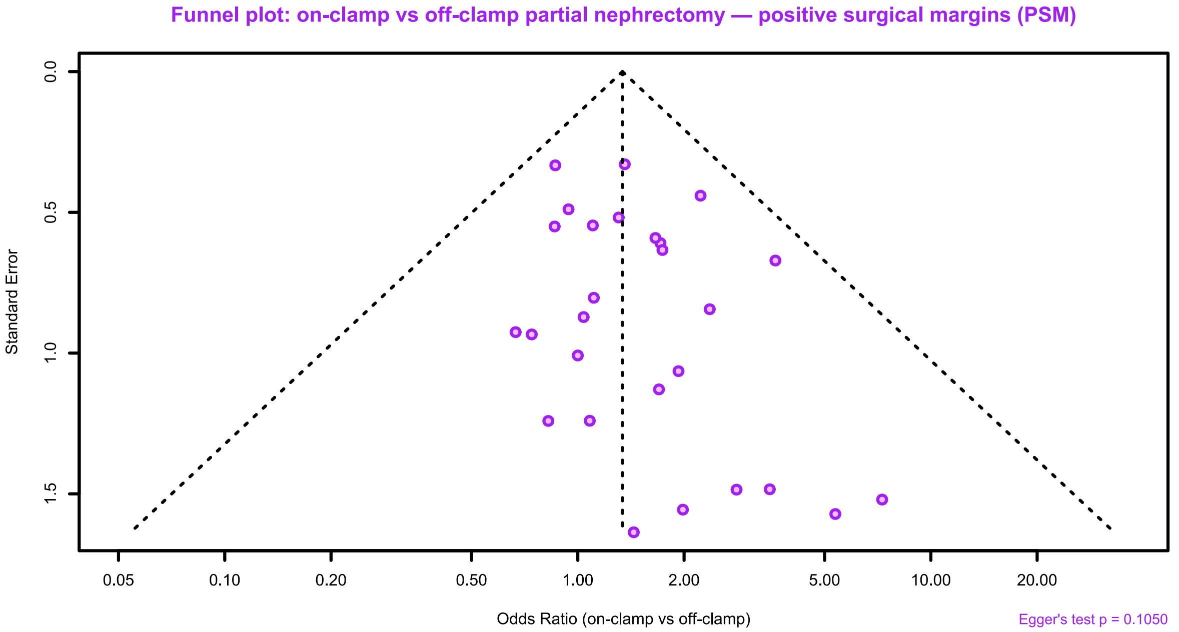

Supplementary Figure S10 - Forest plot presenting the analysis of the operative time between on-clamp and off-clamp partial nephrectomy.

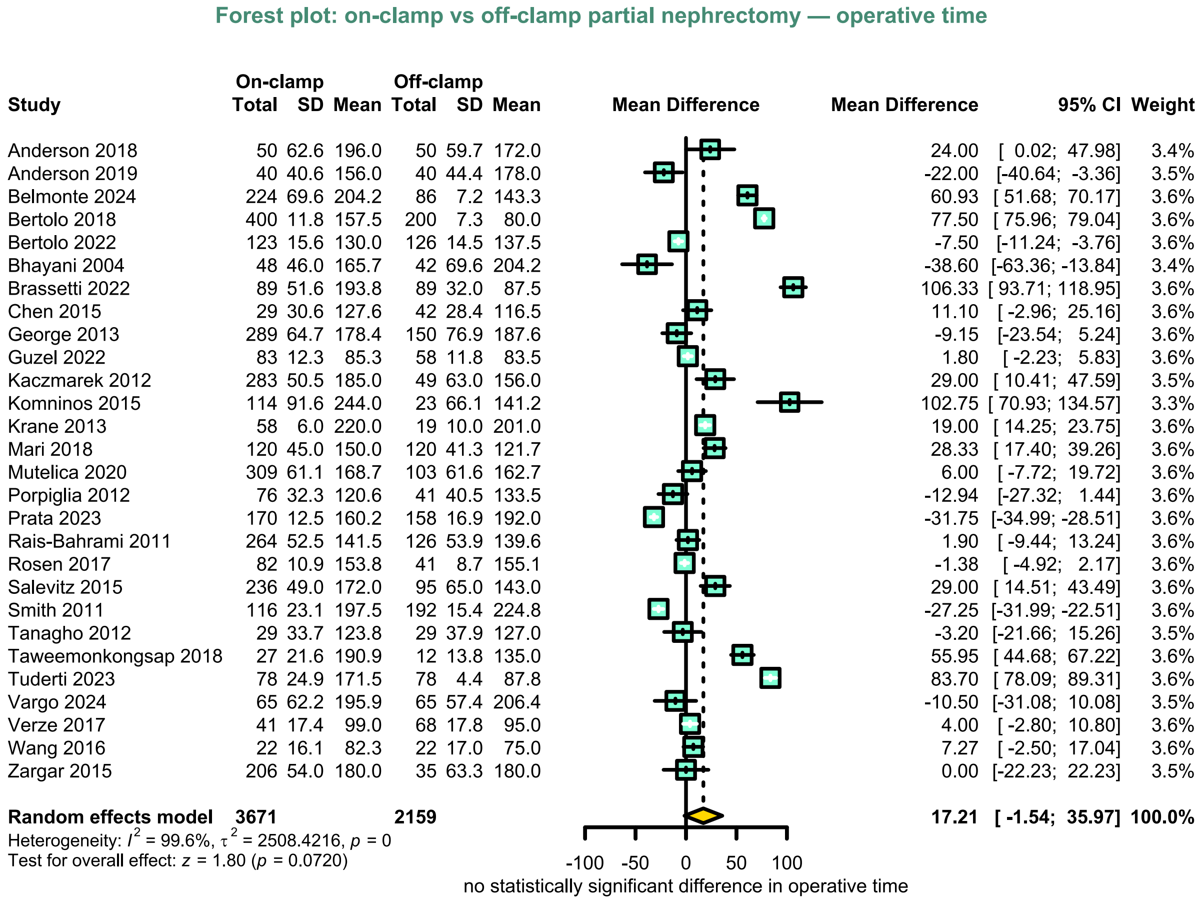

Off-Clamp Versus On-Clamp Partial Nephrectomy: An Updated Systematic Review, Meta-Analysis and Meta-Regression

Table of Content

Supplementary Figure S11 - Forest plot presenting the analysis of the blood transfusion rates between on-clamp and off-clamp partial nephrectomy.

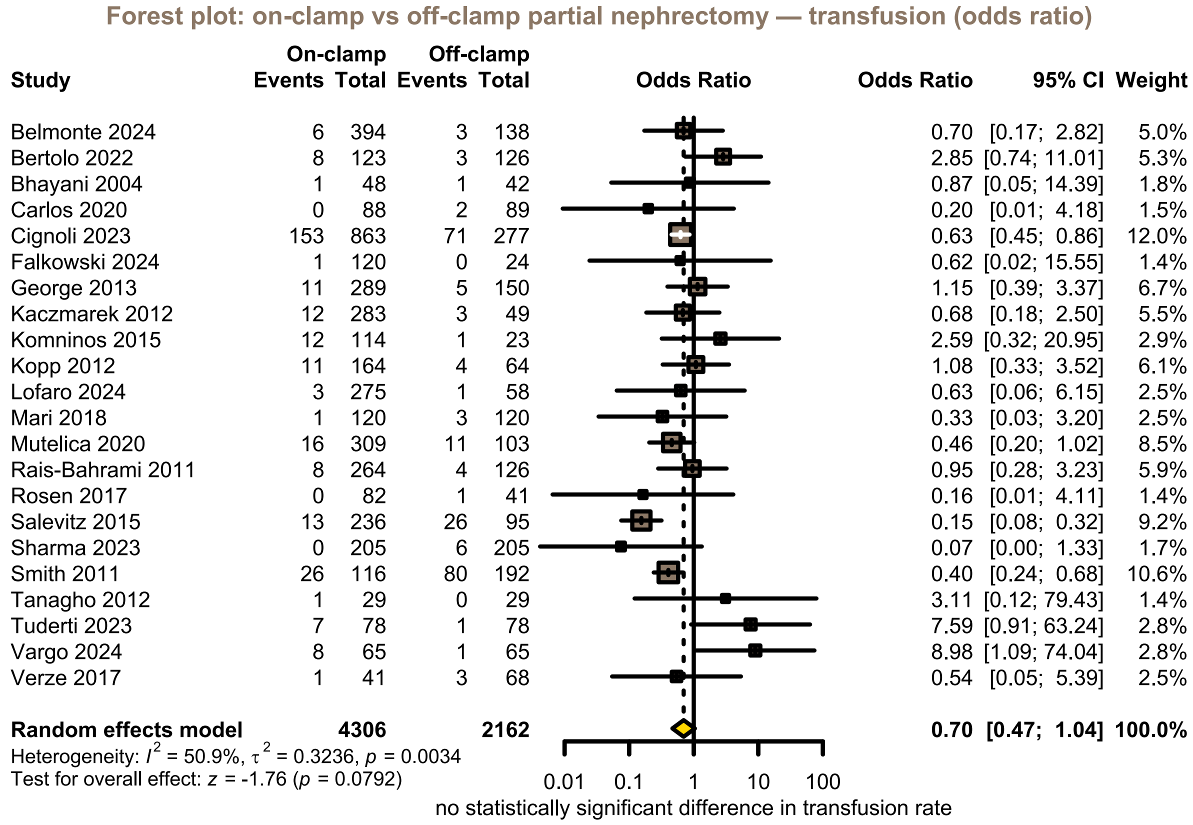

Table of Content

Supplementary Tables

Supplementary Table S1 - Summary of univariable meta-regressions for the association of the estimated glomerular filtration rate (eGFR) changes, % changes in eGFR, and estimated blood loss (EBL) in off-clamp versus on-clamp partial nephrectomy.

Variables		Coefficient	95% CI	*p* value
**eGFR**	Moderator 1	-0.55	-3.9 – 2.8	0.75
	Moderator 2	0.35	-4.4 – 5.1	0.89
	Moderator 3	0.94	-2.8 – 4.7	0.62
	Moderator 4	-0.40	-3.6 – 2.8	0.81
	Moderator 5	0.002	-0.01 – 0.014	0.78
	Moderator 6	-0.14	-1.1 – 0.8	0.77
	Moderator 7	3.42	0.7 – 6.1	**0.014**
	Moderator 8	-0.17	-0.6 – 0.3	0.46
	Moderator 9	-0.007	-0.05 – 0.03	0.73
	Moderator 10	0.10	-0.4 – 0.6	0.72
**% change in eGFR**	Moderator 1	-0.531	-3.7 – 2.7	0.74
	Moderator 2	1.47	-1.0 – 3.9	0.24
	Moderator 3	-1.77	-4.8 – 1.2	0.24
	Moderator 4	0.30	-2.7 – 3.3	0.84
	Moderator 5	-0.005	-0.021 – 0.011	0.56
	Moderator 6	0.26	-1.8 – 2.3	0.80
	Moderator 7	0.90	-2.3 – 4.1	0.58
	Moderator 8	0.11	-0.1 – 0.4	0.37
	Moderator 9	0.03	-0.02 – 0.07	0.27
	Moderator 10	0.18	-0.1 – 0.5	0.28
**EBL**	Moderator 1	1.5	-30.4 – 33.3	0.93
	Moderator 2	-7.4	-60.2 – 45.3	0.78
	Moderator 3	17.2	-52.2 – 86.6	0.63
	Moderator 4	39.2	-13.4 – 91.8	0.14
	Moderator 5	-0.12	-0.22 – -0.01	**0.03**
	Moderator 6	8.9	-7.2 – 25.1	0.28
	Moderator 7	26.1	11.5 – 40.6	**< 0.001**
	Moderator 8	1.0	-3.7 – 5.8	0.67
	Moderator 9	-0.3	-1.0 – 0.4	0.34
	Moderator 10	-0.2	-7.1 – 6.6	0.94

- Moderator 1: surgical technique (RAPN vs LPN vs OPN).
- Moderator 2: time of renal function assessment (<12 months vs ≥12 months).
- Moderator 3: study setting (retrospective vs prospective / RCT).
- Moderator 4: propensity-score matching (no vs yes / RCT).
- Moderator 5: number of patients.
- Moderator 6: tumour size.
- Moderator 7: RENAL nephrometry score.
- Moderator 8: preoperative eGFR.
- Moderator 9: operative time.
- Moderator 10: warm ischaemia time.

CI = confidence interval; eGFR = estimated glomerular filtration rate; EBL = estimated blood loss; RAPN = robot-assisted partial nephrectomy; LPN = laparoscopic partial nephrectomy; OPN = open partial nephrectomy; RCT = randomized controlled trial.

Supplementary Table S2 - The PRISMA 2020 checklist

Section and Topic	Item #	Checklist item	Location where item is reported
**TITLE**			
Title	1	Identify the report as a systematic review.	Title page (Title).
**ABSTRACT**			
Abstract	2	See the PRISMA 2020 for Abstracts checklist.	Abstract (structured).
**INTRODUCTION**			
Rationale	3	Describe the rationale for the review in the context of existing knowledge.	Introduction, paragraphs 1–3 (Section 1).
Objectives	4	Provide an explicit statement of the objective(s) or question(s) the review addresses.	End of Introduction (Section 1; aim statement).
**METHODS**			
Eligibility criteria	5	Specify the inclusion and exclusion criteria for the review and how studies were grouped for the syntheses.	Methods 2.2 Study selection (Eligibility criteria).
Information sources	6	Specify all databases, registers, websites, organisations, reference lists and other sources searched or consulted to identify studies. Specify the date when each source was last searched or consulted.	Methods 2.1 Search strategy (databases searched; last search: Nov 2024).
Search strategy	7	Present the full search strategies for all databases, registers and websites, including any filters and limits used.	Methods 2.1 Search strategy (search terms provided; full database-specific strategies not provided—recommended to add in Supplementary Materials).
Selection process	8	Specify the methods used to decide whether a study met the inclusion criteria of the review, including how many reviewers screened each record and each report retrieved, whether they worked independently, and if applicable, details of automation tools used in the process.	Methods 2.1 Search strategy & 2.2 Study selection (screening by two independent reviewers; disagreements resolved).
Data collection process	9	Specify the methods used to collect data from reports, including how many reviewers collected data from each report, whether they worked independently, any processes for obtaining or confirming data from study investigators, and if applicable, details of automation tools used in the process.	Methods 2.3 Data extraction (two independent extractors; conversions; handling missing SDs).
Data items	10a	List and define all outcomes for which data were sought. Specify whether all results that were compatible with each outcome domain in each study were sought (e.g. for all measures, time points, analyses), and if not, the methods used to decide which results to collect.	Methods 2.2 Study selection (outcomes defined: eGFR, %eGFR, EBL, transfusion, PSM, operative time, complications).
	10b	List and define all other variables for which data were sought (e.g. participant and intervention characteristics, funding sources). Describe any assumptions made about any missing or unclear information.	Methods 2.3 Data extraction & 2.5 Statistical analysis (study/patient variables; moderators for meta-regression).
Study risk of bias assessment	11	Specify the methods used to assess risk of bias in the included studies, including details of the tool(s) used, how many reviewers assessed each study and whether they worked independently, and if applicable, details of automation tools used in the process.	Methods 2.4 Risk of bias and quality assessment; Results 3.1.1; Supplementary Figures 1–2.
Effect measures	12	Specify for each outcome the effect measure(s) (e.g. risk ratio, mean difference) used in the synthesis or presentation of results.	Methods 2.3 Data extraction (MDs/ORs with 95% CIs) and 2.5 Statistical analysis.

Section and Topic	Item #	Checklist item	Location where item is reported
Synthesis methods	13a	Describe the processes used to decide which studies were eligible for each synthesis (e.g. tabulating the study intervention characteristics and comparing against the planned groups for each synthesis (item #5)).	Methods 2.2 Study selection (PICOS; grouping for syntheses by outcomes).
	13b	Describe any methods required to prepare the data for presentation or synthesis, such as handling of missing summary statistics, or data conversions.	Methods 2.3 Data extraction (estimating SDs; converting medians/IQRs to means/SDs).
	13c	Describe any methods used to tabulate or visually display results of individual studies and syntheses.	Methods 2.5 Statistical analysis (forest plots, funnel plots); Results Figures 2–6 and Supplementary Figures 3–11.
	13d	Describe any methods used to synthesize results and provide a rationale for the choice(s). If meta-analysis was performed, describe the model(s), method(s) to identify the presence and extent of statistical heterogeneity, and software package(s) used.	Methods 2.5 Statistical analysis (random-effects model; heterogeneity: Q/I^2; software RStudio/RevMan).
	13e	Describe any methods used to explore possible causes of heterogeneity among study results (e.g. subgroup analysis, meta-regression).	Methods 2.5 Statistical analysis (meta-regression moderators); Results 3.3; Supplementary Table 1.
	13f	Describe any sensitivity analyses conducted to assess robustness of the synthesized results.	Methods 2.5 Statistical analysis (sensitivity analysis described); Results 3.1.1.
Reporting bias assessment	14	Describe any methods used to assess risk of bias due to missing results in a synthesis (arising from reporting biases).	Methods 2.4 Risk of bias and quality assessment (funnel plots, Egger test); Supplementary Figures 3–9.
Certainty assessment	15	Describe any methods used to assess certainty (or confidence) in the body of evidence for an outcome.	Supplementary Materials Table 4
**RESULTS**			
Study selection	16a	Describe the results of the search and selection process, from the number of records identified in the search to the number of studies included in the review, ideally using a flow diagram.	Results 3.1 Study selection and characteristics; PRISMA flow diagram (Figure 1).
	16b	Cite studies that might appear to meet the inclusion criteria, but which were excluded, and explain why they were excluded.	Supplementary Materials Table 2
Study characteristics	17	Cite each included study and present its characteristics.	Results 3.1 and Table 1 (study characteristics).
Risk of bias in studies	18	Present assessments of risk of bias for each included study.	Results 3.1.1; Supplementary Figures 1–2.
Results of individual studies	19	For all outcomes, present, for each study: (a) summary statistics for each group (where appropriate) and (b) an effect estimate and its precision (e.g. confidence/credible interval), ideally using structured tables or plots.	Results Figures 2–6 (forest plots) and Supplementary Figures 10–11 (additional outcomes).
Results of syntheses	20a	For each synthesis, briefly summarise the characteristics and risk of bias among contributing studies.	Results 3.1.1 (RoB summary; sensitivity) and 3.1 (summary of included designs).
	20b	Present results of all statistical syntheses conducted. If meta-analysis was done, present for each the summary estimate and its precision (e.g. confidence/credible interval) and measures of statistical heterogeneity. If comparing groups, describe the direction of the effect.	Results 3.2 Meta-analyses (summary estimates, CIs, heterogeneity) with Figures 2–6 and Supplementary Figures 10–11.
	20c	Present results of all investigations of possible causes of heterogeneity among study results.	Results 3.3 Meta-regression analyses; Supplementary Table 1.

Section and Topic	Item #	Checklist item	Location where item is reported
	20d	Present results of all sensitivity analyses conducted to assess the robustness of the synthesized results.	Results 3.1.1 (sensitivity analysis statement).
Reporting biases	21	Present assessments of risk of bias due to missing results (arising from reporting biases) for each synthesis assessed.	Supplementary Figures 3–9 (funnel plots) and Methods 2.4 (Egger tests).
Certainty of evidence	22	Present assessments of certainty (or confidence) in the body of evidence for each outcome assessed.	Supplementary Materials Table 4
**DISCUSSION**			
Discussion	23a	Provide a general interpretation of the results in the context of other evidence.	Discussion (Section 4; comparison with prior meta-analyses).
	23b	Discuss any limitations of the evidence included in the review.	Discussion—limitations of evidence and included studies (Section 4, 'limitations' paragraph).
	23c	Discuss any limitations of the review processes used.	Discussion—limitations of review processes (Section 4; limitations paragraph—predominance of observational data, heterogeneity, residual confounding).
	23d	Discuss implications of the results for practice, policy, and future research.	Discussion and Conclusions (Sections 4–5; implications for individualized surgical decision-making and future research).
**OTHER INFORMATION**			
Registration and protocol	24a	Provide registration information for the review, including register name and registration number, or state that the review was not registered.	Methods 2.1 Search strategy (PROSPERO registration CRD42024614025).
	24b	Indicate where the review protocol can be accessed, or state that a protocol was not prepared.	Not reported (protocol access link/location not provided).
	24c	Describe and explain any amendments to information provided at registration or in the protocol.	Not reported (amendments not described).
Support	25	Describe sources of financial or non-financial support for the review, and the role of the funders or sponsors in the review.	Funding statement (after Conclusions).
Competing interests	26	Declare any competing interests of review authors.	Conflicts of Interest statement (after Funding).
Availability of data, code and other materials	27	Report which of the following are publicly available and where they can be found: template data collection forms; data extracted from included studies; data used for all analyses; analytic code; any other materials used in the review.	Data Availability Statement (after Conflicts); analytic code not reported as publicly available.

Supplementary Table S3 - Full-text articles excluded, with the main reason for exclusion.

First Author	Year	Journal	DOI	Pubmed_id	Reason for exclusion
Weizer AZ	2008	The Journal of urology	10.1016/j.juro.2008.06.066	18707711	No suture technique in Off Clamp group
Lane B	2010	J. Urol.	10.1016/j.juro.2010.02.1964		Complete data unavailable
Koo HJ	2010	Journal of endourology	10.1089/end.2009.0123	20629571	Cohort of patients less than 30
Lane BR	2011	The Journal of urology	10.1016/j.juro.2010.12.046	21419452	Division into subgroups of ischemia time

Arvanitis I	2011	Eur. Urol. Suppl.	10.1016/S1569-9056(11)61566-2		No change in eGFR or sCr; Complete data unavailable
De Castro Abreu AL	2012	J. Urol.	10.1016/j.juro.2012.02.1893		Complete data unavailable
Petrasz P	2012	Wideochirurgia i inne techniki maloinwazyjne = Videosurgery and other miniinvasive techniques	10.5114/wiitm.2011.30801	23362427	No suture technique
Hung AJ	2013	The Journal of urology	10.1016/j.juro.2012.09.042	23164381	No change in eGFR or sCr
Lamoshi A	2013	J. Endourol.	10.1089/end.2013.2001		Complete data unavailable
Brandao LF	2014	Urology	10.1016/j.urology.2014.04.015	24929947	Cohort of patients less than 30
Springer C	2014	BJU international	10.1111/bju.12376	24053124	Complete data unavailable
Jabaji R	2014	The Canadian journal of urology		24529014	Cohort of patients less than 30
Voylenko O	2014	Urology	10.1016/S0090-4295(14)01020-6		The same study group described in another publication
Porpiglia F	2015	BJU international	10.1111/bju.12834	24913695	Baseline-weighted differential SFR
Morelli L	2015	Journal of Robotic Surgery	10.1007/s11701-015-0519-y		No comparison of warm and no ischemia groups
Shah PH	2016	BJU international	10.1111/bju.13309	26348366	No sCR/eGFR in groups on/off-clamp
Ener K	2016	Turkish journal of urology	10.5152/tud.2016.67790	27909621	Cohort of patients less than 30
Kriegmair M	2016	Eur. Urol. Suppl.			Complete data unavailable
Volkova M	2016	Eur. Urol. Suppl.			Complete data unavailable
Patel M	2016	J. Endourol.	10.1089/end.2016.29020.abstracts		Complete data unavailable
Lieberman L	2017	Journal of endourology	10.1089/end.2016.0678	27936928	No sCR/eGFR in groups on/off-clamp
Ebbing J	2017	J. Urol.			No change in eGFR or sCr,Complete data unavailable
Tang YH	2017	BJU Int.			Complete data unavailable
Ebbing J	2019	BMC nephrology	10.1186/s12882-019-1215-3	30717692	No sCR/eGFR in groups on/off-clamp
Cindolo L	2019	Minerva urologica e nefrologica = The Italian journal of urology and nephrology	10.23736/S0393-2249.18.03357-X	30607928	Complete data unavailable
Antonelli A	2019	The Journal of urology	10.1097/JU.0000000000000194	30827166	The same study group described in another publication
Brassetti A	2019	Eur. Urol. Suppl.	10.1016/S1569-9056(19)31152-2		No full text of the article available
Aquil S	2020	Current urology	10.1159/000499262	32398995	Change in ipsilateral renal function from preoperative
Mercimek MN	2020	Eu. Uro. Op. Sci	10.1016/S2666-1683(20)32640-9		No change in eGFR or sCr; Complete data unavailable;
Lu Qand Zhao X	2020	J. Urol.	10.1097/JU.0000000000000845.04		No comparison of warm and no ischemia groups
Jalbani IK	2020	Pak. J. Med. Sci.	10.12669/pjms.36.3.1533		No change in eGFR or sCr
Guglielmetti GB	2022	The Journal of urology	10.1097/JU.0000000000002695	35404109	No comparison of warm and no ischemia groups
Anceschi U	2022	Minerva Urol. Nephrol.	10.23736/S2724-6051.21.04469-4		Selective ischemia in group on-clamp
Obrecht F	2023	Current oncology (Toronto, Ont.)	10.3390/curroncol30110698	37999118	No sCr or eGFR

Off-Clamp Versus On-Clamp Partial Nephrectomy: An Updated Systematic Review, Meta-Analysis and Meta-Regression

Table of Content

Munoz-Lopez C	2023	BJU international	10.1111/bju.16023	37017637	No sCR/eGFR in groups on/off-clamp
Amparore D	2023	European urology	10.1016/j.eururo.2023.04.005	37117108	No sCR/eGFR in groups on/off-clamp
Flammia RS	2023	Eur. Urol.	10.1016/S0302-2838(23)01070-9		No change in eGFR or sCr,Complete data unavailable
Brassetti A	2024	Eur. Urol.	10.1016/S0302-2838(24)01621-X		T3 only
Cei F	2024	JOURNAL OF UROLOGY			Complete data unavailable

Supplementary Table S4 - GRADE Summary of Findings

**Outcomes**	**No. of participants (studies)**	**Relative effect (95% CI)**	**Anticipated absolute effects / Mean Difference (95% CI)**	**Certainty of the evidence (GRADE)**	**Comments**
**Absolute eGFR change** (mL/min/1.73 m^2)	3,044 (14 studies)	-	**MD 4.2 lower** (5.7 to 2.8 lower)	⊕⊕◯◯ **Low** [a,b]	Favors off-clamp. High heterogeneity.
**Percentage eGFR change** (%)	1,959 (11 studies)	-	**MD 1.7% lower** (2.8% to 0.7% lower)	⊕⊕◯◯ **Low** [a,b]	Favors off-clamp.
**Estimated Blood Loss** (mL)	7,595 (32 studies)	-	**MD 48 higher** (25 to 72 higher)	⊕⊕◯◯ **Low** [a,b]	Favors on-clamp. Significant heterogeneity.
**Postoperative Complications**	6,896 (22 studies)	**OR 1.32** (1.12 to 1.56)	**32 more per 1000** (from 12 to 55 more) [d]	⊕⊕⊕◯ **Moderate** [a]	Favors off-clamp. Homogeneous data.
**Positive Surgical Margins**	7,874 (27 studies)	**OR 1.34** (1.04 to 1.72)	**13 more per 1000** (from 2 to 26 more) [d]	⊕⊕⊕◯ **Moderate** [a]	Favors off-clamp. Homogeneous data.
**Blood Transfusion Rate**	6,468 (22 studies)	**OR 0.71** (0.48 to 1.04)	**11 fewer per 1000** (from 20 fewer to 1 more) [d]	⊕⊕◯◯ **Low** [a,c]	No significant difference.
**Operative Time** (min)	5,830 (28 studies)	-	**MD 17 higher** (1.5 lower to 36 higher)	⊕◯◯◯ **Very Low** [a,b,c]	No significant difference.

Abbreviations: CI, confidence interval; eGFR, estimated glomerular filtration rate; MD, mean difference; OR, odds ratio;

Explanations (GRADE Footnotes):

- **a. Risk of Bias:** Downgraded by one level due to the predominance of observational studies (35 out of 39). While many used propensity score matching, residual confounding cannot be excluded. RCTs also carried "some concerns" regarding blinding.
- **b. Inconsistency:** Downgraded by one level due to high statistical heterogeneity (I^2 > 50% or p < 0.05 for Cochran's Q test).
- **c. Imprecision:** Downgraded by one level as the 95% Confidence Interval crosses the null value (or point of no effect), suggesting uncertainty in the estimate.
- **d. Absolute Effect:** Calculated based on the control group (on-clamp) median event rates observed in the meta-analysis.

Off-Clamp Versus On-Clamp Partial Nephrectomy: An Updated Systematic Review, Meta-Analysis and Meta-Regression

Table of Content

Supplementary Table S5 - Database-specific search strings

Database	Querry	Search Details	Results
PubMed	(partial nephrectomy OR NSS OR nephron sparing surgery) AND (ischemia OR ischemic OR ischemic time OR infarction) AND (function OR insufficiency OR chronic kidney disease OR acute kidney disease OR AKI OR CKD) AND (renal cell cancer OR RCC OR kidney cancer)	((("partial"[All Fields] OR "partials"[All Fields]) AND ("nephrectomy"[MeSH Terms] OR "nephrectomy"[All Fields] OR "nephrectomies"[All Fields])) OR "NSS"[All Fields] OR (("nephronal"[All Fields] OR "nephronic"[All Fields] OR "nephrons"[MeSH Terms] OR "nephrons"[All Fields] OR "nephron"[All Fields]) AND ("spare"[All Fields] OR "spared"[All Fields] OR "spares"[All Fields] OR "sparing"[All Fields]) AND ("surgery"[MeSH Subheading] OR "surgery"[All Fields] OR "surgical procedures, operative"[MeSH Terms] OR ("surgical"[All Fields] AND "procedures"[All Fields] AND "operative"[All Fields]) OR "operative surgical procedures"[All Fields] OR "general surgery"[MeSH Terms] OR ("general"[All Fields] AND "surgery"[All Fields]) OR "general surgery"[All Fields] OR "surgery s"[All Fields] OR "surgerys"[All Fields] OR "surgeries"[All Fields]))) AND ("ischaemia"[All Fields] OR "ischemia"[MeSH Terms] OR "ischemia"[All Fields] OR "ischaemias"[All Fields] OR "ischemias"[All Fields] OR ("ischaemics"[All Fields] OR "ischemia"[MeSH Terms] OR "ischemia"[All Fields] OR "ischaemic"[All Fields] OR "ischemic"[All Fields] OR "ischemical"[All Fields] OR "ischemically"[All Fields] OR "ischemics"[All Fields] OR "ischemized"[All Fields]) OR (("ischaemics"[All Fields] OR "ischemia"[MeSH Terms] OR "ischemia"[All Fields] OR "ischaemic"[All Fields] OR "ischemic"[All Fields] OR "ischemical"[All Fields] OR "ischemically"[All Fields] OR "ischemics"[All Fields] OR "ischemized"[All Fields]) AND ("time"[MeSH Terms] OR "time"[All Fields])) OR ("infarctation"[All Fields] OR "infarcted"[All Fields] OR "infarctic"[All Fields] OR "infarcting"[All Fields] OR "infarction"[MeSH Terms] OR "infarction"[All Fields] OR "infarct"[All Fields] OR "infarctions"[All Fields] OR "infarcts"[All Fields] OR "infarctive"[All Fields])) AND ("functional"[All Fields] OR "functional s"[All Fields] OR "functionalities"[All Fields] OR "functionality"[All Fields] OR "functionalization"[All Fields] OR "functionalizations"[All Fields] OR "functionalize"[All Fields] OR "functionalized"[All Fields] OR "functionalizes"[All Fields] OR "functionalizing"[All Fields] OR "functionally"[All Fields] OR "functionals"[All Fields] OR "functioned"[All Fields] OR "functioning"[All Fields] OR "functionings"[All Fields] OR "functions"[All Fields] OR "physiology"[MeSH Subheading] OR "physiology"[All Fields] OR "function"[All Fields] OR "physiology"[MeSH Terms] OR ("insufficiencies"[All Fields] OR "insufficiency"[All Fields] OR "insufficient"[All Fields] OR "insufficiently"[All Fields]) OR ("renal insufficiency, chronic"[MeSH Terms] OR ("renal"[All Fields] AND "insufficiency"[All Fields] AND "chronic"[All Fields]) OR "chronic renal insufficiency"[All Fields] OR ("chronic"[All Fields] AND "kidney"[All Fields] AND "disease"[All Fields]) OR "chronic kidney disease"[All Fields]) OR (("acute"[All Fields] OR "acutely"[All Fields] OR "acutes"[All Fields]) AND ("kidney diseases"[MeSH Terms] OR ("kidney"[All Fields] AND "diseases"[All Fields]) OR "kidney diseases"[All Fields] OR ("kidney"[All Fields] AND "disease"[All Fields]) OR "kidney disease"[All Fields])) OR "AKI"[All Fields] OR "CKD"[All Fields]) AND ("carcinoma, renal cell"[MeSH Terms] OR ("carcinoma"[All Fields] AND	1006

		"renal"[All Fields] AND "cell"[All Fields]) OR "renal cell carcinoma"[All Fields] OR ("renal"[All Fields] AND "cell"[All Fields] AND "cancer"[All Fields]) OR "renal cell cancer"[All Fields] OR "RCC"[All Fields] OR ("kidney neoplasms"[MeSH Terms] OR ("kidney"[All Fields] AND "neoplasms"[All Fields]) OR "kidney neoplasms"[All Fields] OR ("kidney"[All Fields] AND "cancer"[All Fields]) OR "kidney cancer"[All Fields]))	
Embase	(partial nephrectomy OR NSS OR nephron sparing surgery) AND (ischemia OR ischemic OR ischemic time OR infarction) AND (function OR insufficiency OR chronic kidney disease OR acute kidney disease OR AKI OR CKD) AND (renal cell cancer OR RCC OR kidney cancer)	('partial nephrectomy'/exp OR 'partial nephrectomy' OR (partial AND ('nephrectomy'/exp OR nephrectomy)) OR nss OR 'nephron sparing surgery'/exp OR 'nephron sparing surgery' OR (('nephron'/exp OR nephron) AND sparing AND ('surgery'/exp OR surgery))) AND ('ischemia'/exp OR ischemia OR ischemic OR 'ischemic time'/exp OR 'ischemic time' OR (ischemic AND ('time'/exp OR time)) OR 'infarction'/exp OR infarction) AND ('function'/exp OR function OR insufficiency OR 'chronic kidney disease'/exp OR 'chronic kidney disease' OR (chronic AND ('kidney'/exp OR kidney) AND ('disease'/exp OR disease)) OR 'acute kidney disease'/exp OR 'acute kidney disease' OR (acute AND ('kidney'/exp OR kidney) AND ('disease'/exp OR disease)) OR aki OR ckd) AND ('renal cell cancer'/exp OR 'renal cell cancer' OR (('renal'/exp OR renal) AND ('cell'/exp OR cell) AND ('cancer'/exp OR cancer)) OR rcc OR 'kidney cancer'/exp OR 'kidney cancer' OR (('kidney'/exp OR kidney) AND ('cancer'/exp OR cancer)))	1648
Web of Science	(partial nephrectomy OR NSS OR nephron sparing surgery) AND (ischemia OR ischemic OR ischemic time OR infarction) AND (function OR insufficiency OR chronic kidney disease OR acute kidney disease OR AKI OR CKD) AND (renal cell cancer OR RCC OR kidney cancer)	TS=((partial nephrectomy OR "nephron sparing surgery" OR "nephron-sparing surgery" OR NSS) AND (ischemia OR ischemic OR "ischemia time" OR infarction) AND (eGFR OR "glomerular filtration rate" OR function OR insufficiency OR "chronic kidney disease" OR CKD OR "acute kidney injury" OR AKI) AND ("renal cell carcinoma" OR RCC OR "kidney cancer" OR "renal cancer"))	438
Cochrane Library	(partial nephrectomy OR NSS OR nephron sparing surgery) AND (ischemia OR ischemic OR ischemic time OR infarction) AND (function OR insufficiency OR chronic kidney disease OR acute kidney disease OR AKI OR CKD) AND (renal cell cancer OR RCC OR kidney cancer)	((partial nephrectomy OR NSS OR nephron sparing surgery) AND (ischemia OR ischemic OR ischemic time OR infarction) AND (function OR insufficiency OR chronic kidney disease OR acute kidney disease OR AKI OR CKD) AND (renal cell cancer OR RCC OR kidney cancer)):ti,ab,kw	55
Scopus	(partial nephrectomy OR NSS OR nephron sparing surgery) AND (ischemia OR ischemic OR ischemic time OR infarction) AND (function OR insufficiency OR chronic	TITLE-ABS-KEY ((partial nephrectomy OR NSS OR nephron sparing surgery) AND (ischemia OR ischemic OR ischemic time OR infarction) AND (function OR insufficiency OR chronic kidney disease OR acute kidney disease OR AKI OR CKD) AND (renal cell cancer OR RCC OR kidney cancer))	319

Off-Clamp Versus On-Clamp Partial Nephrectomy: An Updated Systematic Review, Meta-Analysis and Meta-Regression

Table of Content

	kidney disease OR acute kidney disease OR AKI OR CKD) AND (renal cell cancer OR RCC OR kidney cancer)		
